# Northampton General Infirmary

**Published:** 1901-08-17

**Authors:** 


### The Institutional Workshop.

### NORTHAMPTON GENERAL INFIRMARY.

### SPECIAL MEETING OF GOVERNORS.

On Tuesday, 13th inst., a special meeting of the Governors of the Northampton General Infirmary was held for the purpose of considering two schemes for the alteration and improvement of the infirmary buildings. On the 15th of last month, after considering a report by Sir Henry Burdett on the condition of the infirmary, a resolution had been passed authorising the expenditure of £15,000, to be borrowed from the capital fund. The matter had been referred to the committee, which was empowered to again consult Sir Henry Burdett. At Sir Henry's suggestion the assistance of Dr. Richard Greene, late superintendent of Berry Wood Asylum, was also obtained, and Dr. Greene, in connection with Mr. F. W. Dorman, A.R.I.B.A., Northampton, drew up a report in which alternative schemes were submitted to the Governors. Scheme A, it was estimated, could be carried out at a cost of £21,000, while Scheme B was estimated to cost £29,500. It was to consider the report embodying these schemes that Tuesday's meeting was held. The report was as follows:—

### THE REPORT.

#### PRELIMINARY.

We have fully considered the various points submitted at the meeting of the committee on July 6, and we now present a brief description of the two schemes mentioned to the Governors then present. After careful and repeated inspections of the whole infirmary and its surroundings, we have arrived at the conclusion that to place a second storey over the out-patients' department for a nurses' home would be a mistake, and that such second storey, if built, could not be made perfectly suitable for that purpose. Among other objections the space is insufficient, and a third storey would be essential. We doubt whether the foundations of a one-storey building would bear the weight of two superadded storeys; but even if this objection could be got over, we feel sure that the plan is not one which the Governors would approve of when finished.

Having cleared this point we shall describe the salient features of the two schemes which we have worked out, and which we shall designate "Scheme A" and "Scheme B." Plans and estimates of both have been prepared, and will be submitted herewith.

#### Scheme A.

This scheme comprises the erection of a new home for nurses, and certain additions and alterations to the present building. The nurses' home, as planned, consists of a two-storey building, and it seemed to us that the only suitable site for it would be that plot of land lying south-east of the committee-room.

The ground floor contains the kitchen department, the nurses' dining-room and its adjuncts, and rooms for twenty-four nurses. The first floor contains the nurses' sitting-room, reading-room, sick dormitory, and rooms for twenty-four nurses. The sanitary blocks project northwards from each wing of the building, and they are cut off from the main building by properly constructed cross-ventilating passages. Rooms are provided for such domestic servants as would be permanently employed in the nurses' home.

The warming is by open fire-places, assisted where necessary by coils of hot-water pipes on the most approved principle. A corridor would connect the home and the present infirmary.

The remainder of "Scheme A" consists in altering and renovating the greater part of the existing infirmary, and, where necessary, reconstructing it.

It is essential that the sanitary blocks, attached to the wards to the south, be in great part rebuilt. At present they are not properly disconnected from the wards, and are a standing source of danger to the patients. The various fittings in closets, bath-rooms, and sculleries are almost entirely of inferior or obsolete construction.

The wards are not properly provided with ward kitchens, or pantries, or other adjuncts, and to obtain these it will be imperative to divert from their present use the two-bedded rooms in four of the wards. In four of the other wards beds are placed against long stretches of dead wall, where no windows exist, and where no windows could be put. These beds, to the number of 12, would also have to be removed. It would, therefore, follow that a reduction in the number of beds from 150 to, say, 130 as an outside figure, will result from these alterations. In most of the wards it would be necessary to remove the old plaster from the walls and substitute Keen's cement, or adamant, or adamantine.

The floors would have to be entirely relaid with oak or teak.

It is an axiom of correct hospital construction that each bed should have a window on each side of it, and that the windows should run up to the ceiling. It would be possible to lengthen the existing window openings and place louvres in them; but it may not be possible to increase the number of the windows because the construction of the old walls is merely rubble, with an outer and an inner skin; and to cut new openings in such walls may be unsafe. The present ventilators are of the most faulty description, and they must be altogether removed. The system must be altered and amplified.

We estimate the expense of this scheme at about £15,000; but as we have been compelled to sacrifice the two-bedded wards to carry out the alterations, and as we understand that the honorary medical and surgical staff believe strongly in the necessity of these small wards, it would be desirable, perhaps essential, to build a small block on each side of the infirmary containing small wards.

This would entail a further expenditure of £3,000, making the total for "Scheme A" £18,000.

In addition to this, the refurnishing of the hospital and the furnishing of the Nurses' Home would require a sum of about £3,000, making a total expenditure of £21,000.

But this would still leave the infirmary without isolation wards, a microscopic room, a bacteriological laboratory, and wards for diseases of the eye.

#### Scheme B.

This scheme comprises the erection of two new pavilions and the conversion of the present infirmary into a home for the nurses, and a re-arrangement of some of the officers' quarters.

The new pavilions would be of three storeys; and these blocks would be connected with each other by a one-storey corridor running east and west, and this would be joined at right angles by another, which would spring from the present surgery, and which would run north and south. There would be a subway from the kitchen to the corridor, and as the difference in level is only about one in twenty, a tram-line for the dinner wagons could be easily coestructed, and the food quickly distributed to the various wards. The staircases and lifts are placed north of the pavilions, and from these staircases run covered bridges connecting the new with the old buildings.

The new pavilions are of the most modern and complete description. They will include large wards, verandahs, fire escapes, four-bedded wards, single-bedded wards, and ward kitchens; pantries, linen-rooms, patients' clothes stores, coal stores, examining rooms, etc., etc. The sanitary blocks are separated from the wards by cross-ventilated passages.

The warming is by open fire-places, supplemented, where necessary, by hot-water coils.

The lighting will be by electricity, and electric fans for the extraction of impure air will be placed in the wards.

Fresh air will be admitted on the floor line by a new principle, which permits the valve to be drawn out, cleaned, and replaced in a minute. Five beds on each side would be fitted up for diseases of the eye. In all instances the walls and floors would be made as nearly impervious as possible.

The present infirmary would be arranged somewhat as follows:—On the north side of central corridor, and turning to the right on entering the infirmary, would be the secretary's office and an office for the secretary's clerk. Next to these would be retiring rooms for the honorary physicians. On the right on entering would be the hall-porter's room, and bedroom, a store-room, and a common sitting-room for the resident medical staff. On the south side of this central corridor would be the sitting-room and bedroom for the senior house surgeon, rooms for the junior house surgeon, and rooms for the resident dresser.

The operating theatre would be untouched (except as hereafter described), and close to it would be the retiring-rooms for the honorary surgeons. A new passage leads from the operating theatre corridor to the new accident ward, the said passage occupying part of the present ward, while the remaining part would be converted into an opthalmoscopic room, a room for the electric medical batteries, and a room for surgical appliances.

The ground floor of the west wing would be converted into an isolation ward for women. It is rather surprising that no isolation ward exists at present. This ward has ample space for four beds, and there would be a small ward attached for a single patient. If the plan be referred to, it will be noticed that this ward is provided with a passage and double doors, and that any of the kitchen staff approaching it would pass along another corridor and deposit the food in the ward, kitchen, or scullery, next to the nurses' duty-room, so that there need be no inter-communication of the kitchen staff with the nursing staff.

The present medical library would be converted into a sitting-room for the lady superintendent of nurses. A store-room would be attached, and a short corridor would communicate with the main corridor, enabling the superintendent of nurses to be in any part of the hospital without delay.

This is perhaps the best place to name the purpose for which the floor over the committee-room is intended. On one side would be the medical library, and the other would be fitted up as a pathological museum, a microscope room, and a laboratory for bacteriological investigations. No Infirmary can nowadays be considered properly equipped if it be deficient in these adjuncts.

On the first floor of the main building (in the centre) would be the nurses' sitting-room, reading-room, and cloak-room, and the nurses' dining-room, the latter having attached to it a scullery, a linen-room, a store-room, and a food lift from the kitchen.

The first floor of the main building would provide bedrooms for 16 nurses, together with bath-rooms, closets, box-rooms, and store-rooms.

The Victoria Ward on this floor would remain practically untouched, it being the only ward in the old building which approaches to correct hospital construction. This ward would be known as the dormitory for sick nurses, an adjunct which every modern infirmary now possesses. It is one which has a decided tendency to reduce the number of days lost by the nursing staff in sickness. It is true that this ward is somewhat larger than is absolutely necessary, except, perhaps, in an influenza or other epidemic; but in summer when there is but little sickness among the nurses, the ward would be found very useful as a temporary or transfer ward when any ward in the new pavilion was being "done up" or thoroughly "spring cleaned."

We have shown our method of providing our isolation ward for the women; and we can obtain a similar one for the men by putting a storey on the operating theatre. This would in no way interfere with the proper use of the latter. The floors would be thoroughly "deadened" and "fire-proofed" so that no sound could penetrate. The present ceiling would not be touched, and during construction it would be protected from the weather by a waterproof tent.

On the second floor of the old building provision for thirty nurses and fourteen of domestic and laundry staff. The cook and the head laundress would have separate bedrooms.

We wish to draw the attention of the Governors to the following points in connection with "Scheme B."

1. The new blocks can be erected without disturbing the present wards.
2. The new wards are absolutely distinct from the administrative department, and at the same time communication exists between every floor of the new with every floor of the old by means of covered bridges.
3. Every part of the present building is utilised.
4. The cost of "keeping up" would be somewhat less than that of the other scheme.
5. Without reckoning the isolation wards or the sick nurses' dormitory the accommodation would be for 126 beds.

The estimated expense of "Scheme B," including furniture is about £29,500.

But if the men's isolation ward be omitted, deduct £550 for the second storey over present operating theatre.

(Signed) RICHARD GREENE.
(Signed) FRED. W. DORMAN.

July 27th, 1901.

At a meeting of the Special Joint Committee on July 27th, 1901, Sir Henry Burdett and Dr. Greene made a joint offer of £1,000 provided Scheme B was adopted by the Governors, and on the condition that nine other donations of £1,000 each were forthcoming, thus making £10,000, which is practically the difference between Scheme A and Scheme B.

At Tuesday's meeting the chair was taken by the Rev. L. H. Loyd, who called upon Sir Henry Burdett to give a detailed explanation of the plans. Sir Henry Burdett said that the difference between the cost of the two schemes (£10,000) would represent the difference between a new hospital which they would be proud of for a century and a hospital which, as a matter of fact, would not last them probably more than 20 years, or, at any rate, would not last them with satisfaction for a much longer period. As the result of his efforts, with the invaluable help of local gentlemen, he was able to place on the table a list of promises of donations amounting to £10,700 towards the difference between the cost of Scheme A, to which the Governors were practically committed, and the better Scheme B. A thousand pounds each had been promised by the following:—Lord Spencer, Lady Wantage, Mr. W. H. Foster, J.P., the Marquis of Northampton, Mrs. Pickering Phipps and Miss Phipps, the Mayor of Northampton, Mr. J. Cooper, J.P., Messrs. Phipps and Co., and Mr. H. Manfield, J.P. Lord Lilford promised £500, Mr. David Dulley promised £100, and a donation of £100 in cash had already been received. He had reason to hope that his efforts would result in the raising of £15,000. After making an earnest and eloquent appeal for the larger scheme, Sir Henry read letters supporting it from Lord Spencer, Lord Northampton, and others who had promised £1,000.

The Chairman moved the adoption of the report of the Special Joint Committee, which briefly recommended to the Governors the adoption and carrying out substantially of Scheme B.

Sir Henry, in reply to Mr. Markham, said that so far as he could make out the care which had been exercised in the plans warranted him in believing that the £30,000 estimate for the cost of Scheme B could be relied upon.

Dr. Greene, also replying to Mr. Markham's question, said he believed £30,000 was an outside estimate, and thought the actual cost would be less than that sum.

Dr. Hollis proposed that the question be adjourned for six months, and that Messrs. Young and Hall be called in to advise. He contended that the absolute necessity for this gigantic scheme had not yet been proved.

After some further discussion, the amendment was lost by a large majority, and the report of the committee recommending the adoption of Scheme B was carried *nem. con.*

A full report of the discussion appeared in Wednesday's *Northampton Daily Reporter.*

